# A Five-Genes Based Diagnostic Signature for Sepsis-Induced ARDS

**DOI:** 10.3389/pore.2021.580801

**Published:** 2021-07-29

**Authors:** Ning Xu, Hui Guo, Xurui Li, Qian Zhao, Jianguo Li

**Affiliations:** ^1^ Department of Emergency, Hebei General Hospital, Shijiazhuang, China; ^2^ Department of General Practice, Hebei General Hospital, Shijiazhuang, China

**Keywords:** sepsis, ARDS, GO and KEGG analysis, logistic regression model, SVM model

## Abstract

**Background:** Acute respiratory distress syndrome (ARDS) is a frequent and serious complication of sepsis without specific and sensitive diagnostic signatures.

**Methods:** The mRNA profiles, including 60 blood samples with sepsis-induced ARDS and 86 blood samples with sepsis alone, were obtained from the Gene Expression Omnibus (GEO). The differently expressed genes (DEGs) were analyzed by *limma* package of R language. Gene Ontology (GO) analysis and Kyoto Encyclopedia of Genes and Genomes (KEGG) pathway enrichment analysis were carried out using the *clusterProfiler* package of R. Eventually, multivariate logistic regression model was established through the *glm* function of R, and support vector machine (SVM) model was constructed via the *e1071* package of R.

**Results:** A total of 242 DEGs in GSE32707 and 102 DEGs in GSE66890 were identified. Notably, five genes exhibited significant differences between the two datasets and were considered to be closely associated with the occurrence of ARDS induced by sepsis. Furthermore, functional enrichment analysis based on the DEGs showed there were 80 overlapped GO terms and one KEGG pathway which were significantly enriched in the two datasets. The logistic regression model and SVM model constructed could efficiently distinguish sepsis patients with or without ARDS.

**Conclusion:** In brief, our study suggested that NKG7, SPTA1, FGL2, RGS2, and IFI27 might be potential diagnostic signatures for sepsis-induced ARDS, which contributed to the future exploration in mechanism of ARDS occurrence and development.

## Introduction

Acute respiratory distress syndrome (ARDS) is a non-cardiogenic form of pulmonary oedema caused by alveolar injury secondary to an inflammatory process, and is significantly characterized by refractory hypoxemia [1]. ARDS makes up 10% of intensive care unit admissions, representing over three million patients with ARDS worldwide each year [2]. Since its first description, ARDS has been recognized as a main clinical challenge because of the high morbidity and mortality in respiratory medicine [3]. Lung injury is a common disease in sepsis, and could lead to the severe complication of ARDS [4]. Although the pathogenic factors of ARDS are various, it is commonly caused by sepsis due to non-pulmonary sources, severe trauma, and aspiration of gastric contents [5]. Severely, the ARDS induced by sepsis shows increasing incidence and higher mortality compared with the ARDS induced by other factors [6, 7]. The Berlin Clinical Classification defined ARDS according to PaO_2_/FiO_2_ ratio and bilateral infiltrates as clinical criteria [8]. Nevertheless, the clinical criteria could hardly guide treatment. Besides, due to the heterogeneity between individuals, diagnostic criteria is difficult to be related to pathogenesis [9]. Therefore, it is imperative to identify specific biomarkers for the diagnosis of ARDS or sepsis-induced ARDS.

Recently, more attentions have been paid to sepsis-induced ARDS and the specific pathogenic mechanism is also well studied, meanwhile, many potential genes associated with the occurrence and progression of the ARDS induced by sepsis have been identified. For instance, Kangelaris et al. found that the important mediators of the initial neutrophil response to infection, including olfactomedin 4, lipocalin 2, CD24, and bactericidal/permeability-increasing protein were obviously and differentially expressed between patients with sepsis complicated with ARDS and patients with sepsis alone, suggesting that these genes were potentially associated with the pathogenesis of ARDS related to sepsis [10]. Zhang et al. demonstrated that the transcription factors MYC and STAT3 might play a regulatory role in the underlying dysfunction of ARDS induced by sepsis, and receiver operating characteristic (ROC) curve analysis revealed MYC and STAT3 might be considered as significant markers for sepsis or sepsis-induced ARDS [11]. S100A12, a pro-inflammatory factor, can promote inflammation and cell apoptosis in ARDS induced by sepsis through activating the NLRP3 inflammasome signaling pathway, which is a potential biomarker of pulmonary injuries in the clinical diagnosis of ARDS induced by sepsis [12]. Xue et al. found that patients with ARDS induced by sepsis exhibited markedly increased median levels of tissue factors than those with sepsis alone, and indicated that tissue factor was a valuable diagnostic and prognostic biomarker for the ARDS induced by sepsis [13]. These evidences indicated that the key genes which showed differential expression between patients with sepsis-induced ARDS and those with sepsis alone might be potential diagnostic signatures.

In this study, five key genes including NKG7, SPTA1, FGL2, RGS2, and IFI27 displayed obvious differences in blood samples with sepsis-induced ARDS in comparison to those with sepsis alone in the two datasets, which suggested that these five genes were probably associated with the development of sepsis-induced ARDS. Furthermore, the classification model including logistic regression model and SVM model established based on the five key genes could efficiently distinguish samples with sepsis-induced ARDS from those with sepsis alone. Our results indicated that the five key genes were potential biomarkers for sepsis-induced ARDS, which could be helpful for better understanding of ARDS occurrence and development.

## Materials and Methods

### Datasets

The mRNA profiles of GSE32707 [14] and GSE66890 [10] were obtained from Gene Expression Omnibus (GEO, https://www.ncbi.nlm.nih.gov/geo/). GSE32707 included 31 blood samples with sepsis-induced ARDS and 58 blood samples with sepsis alone, and GSE66890 included 29 blood samples with sepsis-induced ARDS and 28 blood samples with sepsis alone. The mRNA profiles of GSE32707 were determined by Illumina HumanHT-12 V4.0 expression beadchip, and the mRNA profiles of GSE66890 were examined by Affymetrix Human Gene 1.0 ST Array. Besides, the detailed clinical information of samples from these two datasets was shown in Supplementary Table S1 and previous research [14].

### Differential Expression Analysis

We removed the probes of mRNA profiles with missing value, and conducted the standardization by using robust multi-array (RMA) method. Subsequently, we performed the differential expression analysis of genes by the *limma* package of R language [15], with |log 2 (fold change [FC])| > 0.5 and *p* < 0.05 as the significant thresholds.

### Functional Enrichment Analysis

Gene Ontology (GO) analysis which included biological process, molecular function and cellular component, and Kyoto Encyclopedia of Genes and Genomes (KEGG) pathway enrichment analysis were carried out by using the *clusterProfiler* package of R language [16], with *p* < 0.05 as the significant threshold.

### Construction of Logistic Regression Model and Support Vector Machine (SVM) Model

The multivariate logistic regression model was constructed by using the *glm* function of R language [17], in which the expression value of genes was used as the continuous predictive variable and the sample type (sepsis with or without ARDS) was used as the categorical responsive value. Meanwhile, the *e1071* package of R language (https://cran.r-project.org/web/packages/e1071/index.html) was applied to construct the SVM model. In this model, the expression value of genes was used as the continuous predictive variable and the sample type (sepsis with or without ARDS) was used as the categorical responsive value. Subsequently, *caret* package (https://CRAN.R-project.org/package=caret) of R language was used for 5-fold cross-validation, and the reliability of the model was evaluated according to the area under curve (AUC) value of the receiver operating characteristics (ROC) curve.

## Results

### Identification of Differentially Expressed Genes

**FIGURE 1:**
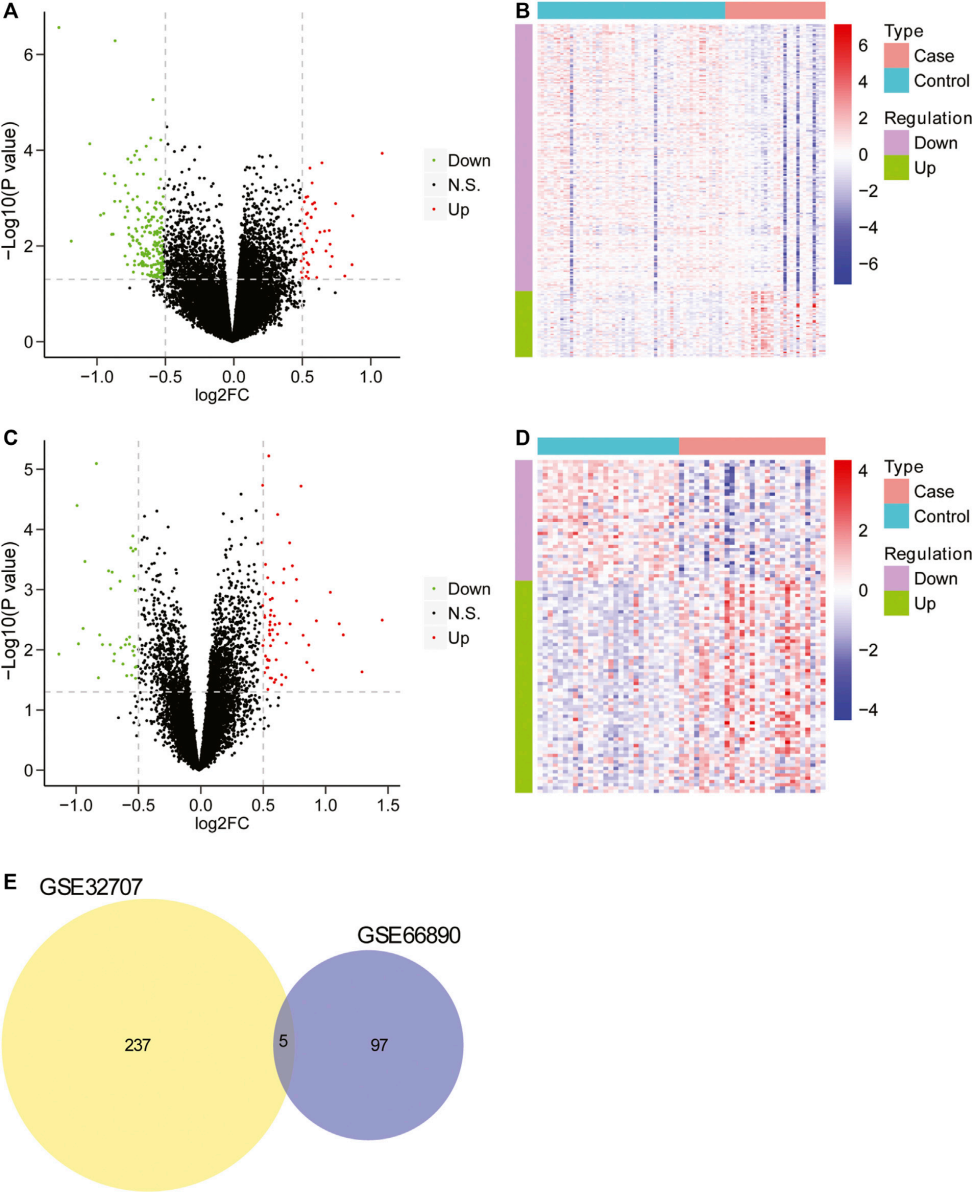
The screening of differentially expressed genes. **(A)** The volcano plot of differentially expressed genes between two groups in GSE32707. The horizontal axis was Log2 FC, and the vertical axis was −log10 (*p* value). The red points represented upregulated genes, the green points represented downregulated genes, and the black points indicated no obvious difference. **(B)** The heatmap of differentially expressed genes between two groups in GSE32707. The horizontal axis represented genes, the vertical axis represented samples, red indicated high expression, blue indicated low expression. **(C)** The volcano plot of differentially expressed genes between two groups in GSE66890 dataset. The horizontal axis was Log2 FC, and the vertical axis was −log10 (*p* value). The red points represented upregulated genes, the green points represented downregulated genes, and the black points indicated no manifest difference. **(D)** The heatmap of differentially expressed genes between two groups in GSE66890 dataset. The horizontal axis represented genes, the vertical axis represented samples, red indicated high expression, and blue indicated low expression. **(E)** The venn diagram of differentially expressed genes in datasets GSE32707 and GSE66890.

Firstly, the data from GSE32707 and GSE66890 datasets was standardized and the results revealed that there was no distinct difference in the overall distribution of mRNA expression for each sample from these two datasets (Supplementary Figure S1), suggesting that the data could be used for subsequent analysis. Then the differential expression analysis was performed in GSE32707 dataset, and 242 differentially expressed genes were identified in the group with sepsis-induced ARDS in comparison to that with sepsis alone, which consisted of 48 upregulated genes and 194 downregulated genes (Figure 1A), and the expression of 242 genes exhibited marked difference between the two groups (Figure 1B); In GSE66890 dataset, we identified 102 differentially expressed genes between the group with sepsis-induced ARDS and the group with sepsis alone, consisting of 65 upregulated genes and 37 downregulated genes (Figure 1C), and the expression of 102 genes displayed obvious difference between the two groups (Figure 1D). Moreover, five genes including NKG7, SPTA1, FGL2, RGS2, and IFI27 exhibited notable difference between the sepsis-induced ARDS group and the control group with sepsis alone in two datasets (Figure 1E), suggesting that these five genes might be key genes that led to sepsis patients complicated with ARDS.

### Functional Enrichment Analysis

**FIGURE 2:**
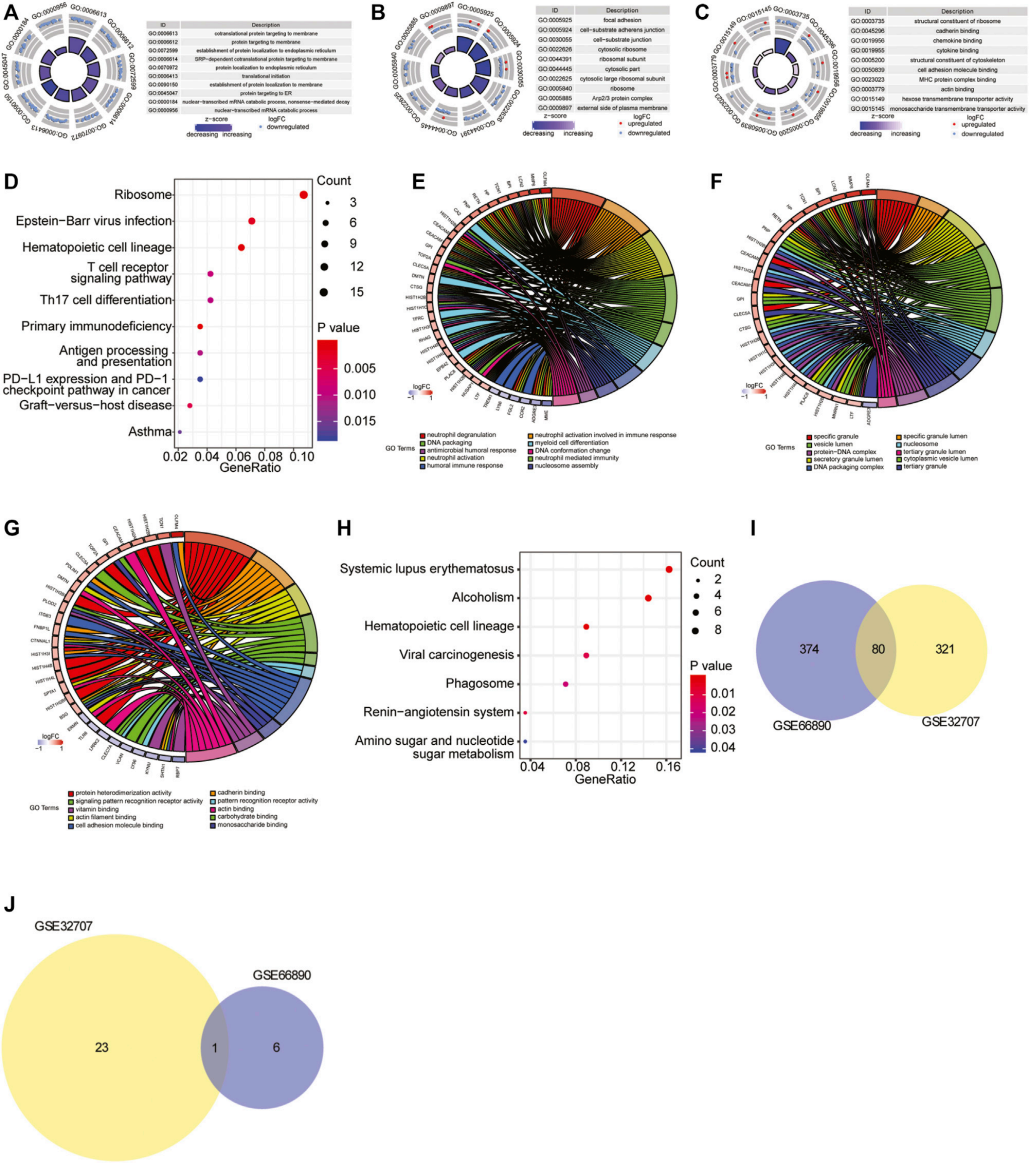
GO and KEGG pathway enrichment analysis. **(A–C)** The top 10 most significantly enriched BP **(A)**, CC **(B)** and MF **(C)** terms in GSE32707 dataset. In GO terms, each point in the ring represented a mRNA, red indicated the gene with increased expression, and blue indicated the gene with decreased expression; the color of the innermost ring indicated z-score, and the darker purple indicated the more significant enrichment result. **(D)** The top 10 most significantly enriched KEGG pathways in GSE32707 dataset. The horizontal axis represented GeneRatio (enrichment ratio), the vertical axis indicated the biological process or KEGG pathway. The size of the dot represented the number of genes that were enriched, and the dot color indicated *p* value. **(E–G)** The top 10 most significantly enriched BP **(E)**, CC **(F)** and MF **(G)** terms in GSE66890 dataset. The right half circle indicated the 10 BP terms represented by altered colors. The left half circle indicated the gene enriched in the 10 terms. Red indicated up-regulated gene, and blue indicated down-regulated gene. **(H)** The top seven most significantly enriched KEGG pathways in GSE66890 dataset. The horizontal axis represented GeneRatio (enrichment ratio), the vertical axis indicated the corresponding biological process or KEGG pathway. The size of the dot represented the number of genes that were enriched, and the dot color represented *p* value. **(I)** The venn diagram of overlapped enriched GO terms both in GSE32707 and GSE66890 datasets. **(J)** The venn diagram of overlapped enriched KEGG pathways both in GSE32707 and GSE66890 datasets.

To investigate the biological processes and pathways closely involved in sepsis-induced ARDS, the functional enrichment analysis was performed based on the differentially expressed genes of two datasets. For GSE32707 dataset, there were 274 significantly enriched biological process (BP) terms including immune response, neutrophil activation and T cell activation (*p* < 0.05), 78 significantly enriched cellular component (CC) terms including cell-substrate adherens junction, cell-cell junction and receptor complex (*p* < 0.05), 49 significantly enriched molecular function (MF) terms including cadherin binding, cell adhesion molecule binding and cadherin binding (*p* < 0.05), and 24 significantly enriched KEGG pathways including Epstein-Barr virus infection, T cell receptor signaling pathway and antigen processing and presentation (*p* < 0.05). The full list of those significantly enriched GO terms and KEGG pathways were shown in Supplementary Table S2. In addition, the top 10 most significantly enriched BP, CC and MF terms were displayed in Figures 2A–C, and the top 10 most significantly enriched KEGG pathways were exhibited in Figure 2D. For GSE66890 dataset, there were 370 significantly enriched BP terms including neutrophil degranulation/activation, neutrophil mediated immunity and DNA packaging (*p* < 0.05), 62 significantly enriched CC terms including secretory granule lumen and cytoplasmic vesicle lumen (*p* < 0.05), 22 significantly enriched MF terms including protein heterodimerization activity, cadherin binding and actin filament binding (*p* < 0.05), and seven significantly enriched KEGG pathways including systemic lupus erythematosus, phagosome and hematopoietic cell lineage (*p* < 0.05). The full list of significantly enriched GO terms and KEGG pathways were shown in Supplementary Table S3. The top 10 most significantly enriched BP, CC and MF terms were displayed in Figures 2E–G, and seven most significantly enriched KEGG pathways were exhibited in Figure 2H. Moreover, there were 80 overlapped GO terms (Figure 2I) and one overlapped KEGG pathway (hematopoietic cell lineage) (Figure 2J) which were significantly enriched between two datasets, and the full list of the overlapped GO terms and KEGG pathway was shown in Supplementary Table S4. Based on the above analyses, we speculated that the overlap in significantly enriched GO terms and KEGG pathway might represent vital pathways in the sepsis-induced ARDS development.

### Construction of Classification Model

**FIGURE 3:**
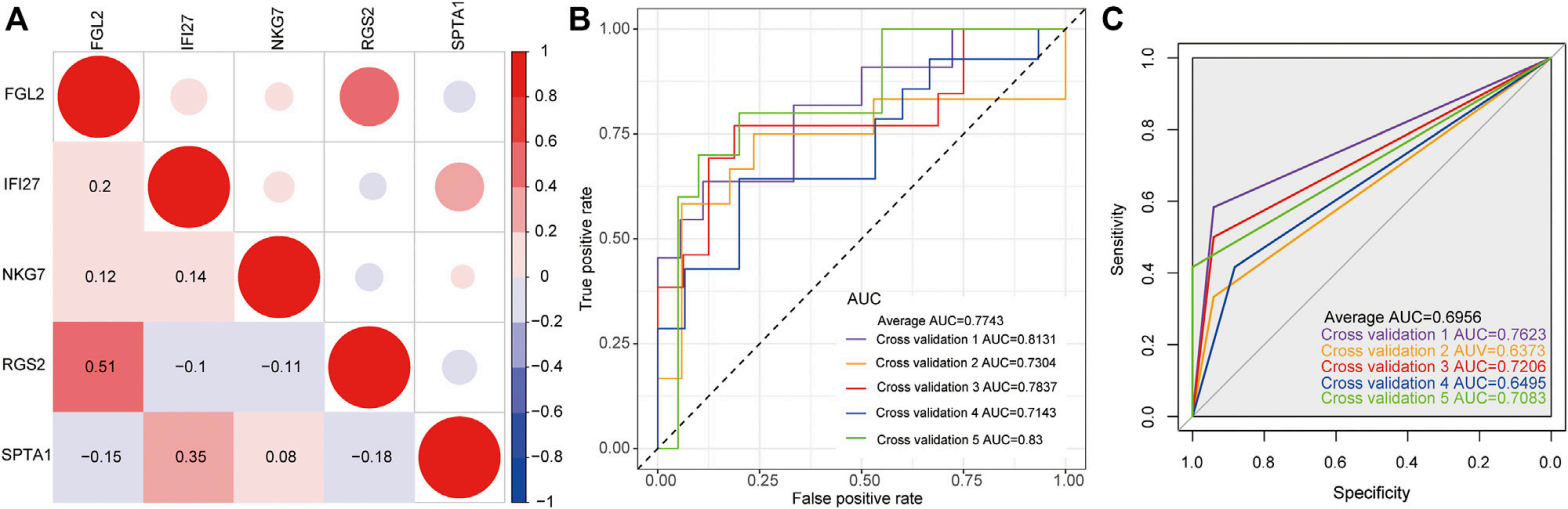
The construction of classification model. **(A)** The correlation matrix of five key genes. Red represented positive correlation, while blue represented negative correlation. Darker color indicated greater correlation. **(B)** The ROC curve of logistic regression model by 5-fold cross-validation. **(C)** The ROC curve of SVM model by 5-fold cross-validation.

The expression levels of five key genes (NKG7, SPTA1, FGL2, RGS2, and IFI27) in two datasets of GSE32707 and GSE66890 were analyzed by *sva* package of R language to eliminate the batch effect. Correlation analysis of the five genes expressions was conducted. (Figure 3A). The pairings of these five genes were too weakly correlated to be removed. Then 5-fold cross-validation was constructed to verify the reliability of the model. As shown in Figure 3B, the ROC curve was the logistic regression model with 5-fold cross-validation. The results indicated that the AUCs of the 5-fold cross-validation were 0.8131, 0.7304, 0.7837, 0.7143, and 0.83, respectively, and the average AUC was 0.7743. Meanwhile, the expression level of these five genes was used as a continuous variable and the sample type (sepsis with or without ARDS) as a categorical responsive value, then the SVM model was constructed. The ROC curve of the 5-fold cross-validation for SVM model were displayed in Figure 3C. The AUCs were 0.7623, 0.6373, 0.7206, 0.6495, and 0.7083, respectively, and the average AUC was 0.6956. The above results revealed that the logistic regression model and SVM model based on NKG7, SPTA1, FGL2, RGS2, and IFI27 could efficiently distinguish samples with sepsis and ARDS from samples with sepsis alone.

## Discussion

Although the treatment of sepsis is very effective in the last decades [18], exploring the potential mechanism of sepsis-induced ARDS and identifying more specific and sensitive signatures for clinical diagnosis are still necessary. Our perceiving of ARDS is still at the clinical diagnostic level, and we haven’t had a comprehensive understanding of the pathological mechanism. Recent interventions have focused on improving oxygenation and avoiding iatrogenic loss, rather than direct treatment. With the deepening of research, we could not only screen auxiliary diagnostic markers, but also fully utilize them to better guide the treatment. Herein, 242 and 102 differentially expressed genes in the two datasets were identified based on the whole blood gene expression profiles of sepsis patients with or without ARDS. Then the functional enrichment analysis revealed that there were 80 significantly overlapped enriched GO terms and one overlapped KEGG pathway (hematopoietic cell lineage) between the two datasets. The enriched GO terms majorly included neutrophil activation, positive regulation of leukocyte cell-cell adhesion, T cell activation and cellular defense response and so on, suggesting that immune response might account for the development of sepsis-induced ARDS. Previous studies have demonstrated that ARDS was an acute inflammatory lung injury caused by sepsis or other factors [19, 20]. As numerous cytokines could facilitate ARDS progress, including the pathological and physiological processes, it is suggested that inflammatory response is closely involved in ARDS pathogenesis [21]. Previous study demonstrated that simultaneous production of inflammatory cytokines was implicated in the inflammatory process of acute lung injury induced by sepsis [22]. Besides, Khatri et al. identified some clusters based on gene expression, including termed inflammopathic according to the transcriptomic data of sepsis patients [23]. Our results confirmed that most enriched inflammatory response-related biological processes were consistent with previous evidences, and provided theoretical basis for the subsequent analysis.

Furthermore, five genes including NKG7, SPTA1, FGL2, RGS2 and IFI27 exhibited significant differences between the sepsis-induced ARDS samples and the samples with sepsis alone in two datasets, suggesting that these five genes might be key genes that led to sepsis patients complicated with ARDS. Although the effects of these five genes have not been studied in sepsis-induced ARDS, their specific roles in inflammatory response are well known. NKG7 is expressed in natural killer cells and T cells, and closely involved in host-defense mechanisms against infection and cancer, as well as the immune response regulation [24]. SPTA1 is involved in the mutually exclusive gene set, and mutually exclusive with cell cycle members, P53 and RB pathways, and mutated SPTA1 might be associated with the development of glioblastoma [25]. Fan et al. demonstrated that in human idiopathic pulmonary arterial hypertension, fibrinogen-like protein 2 (FGL2) participated in the pathological progression of pulmonary hypertension (PH) [26]. RGS2 is a negative regulator of STAT3-mediated Nox1 expression, which is essential for the production of reactive oxygen species in the innate immune response [27]. Interferon α-inducible protein 27 (IFI27) is involved in innate immunity and the elevated expression of IFI27 could enhance the proliferation, migration, and invasion of cells in cholangiocarcinoma [28]. These data indicated that these five genes were remarkably involved in the immune-related diseases or cancers, and might be associated with the occurrence of ARDS induced by sepsis.

Then we established the logistic regression model and SVM model with the five key genes to distinguish sepsis patients with or without ARDS. These results revealed that our classification model showed potential application in the early diagnosis of sepsis-induced ARDS. Nevertheless, there were some limitations in this study: 1) more samples would be helpful to determine the accuracy of the classification model; 2) the specific roles of these five genes should be studied in detail.

## Conclusion

In summary, our study identified five key genes including NKG7, SPTA1, FGL2, RGS2, and IFI27, which were closely related to the sepsis-induced ARDS development. The classification model with the five genes could efficiently discriminate sepsis patients with or without ARDS, suggesting that these key genes might be potential diagnostic signatures for sepsis-induced ARDS.

## Data Availability

The datasets presented in this study can be found in online repositories. The names of the repository/repositories and accession number(s) can be found in the article/Supplementary Material.

## References

[1] Sweeney RM McAuley DF. Acute Respiratory Distress Syndrome. The Lancet (2016) 388(10058):2416–30. 10.1016/s0140-6736(16)00578-x PMC7138018 27133972

[2] Fan E Brodie D Slutsky AS. Acute Respiratory Distress Syndrome. JAMA (2018) 319(7):698–710. 10.1001/jama.2017.21907 29466596

[3] Confalonieri M Salton F Fabiano F. Acute Respiratory Distress Syndrome. Eur Respir Rev (2017) 26(144):160116. 10.1183/16000617.0116-2016 28446599 PMC9488505

[4] Englert JA Bobba C Baron RM. Integrating Molecular Pathogenesis and Clinical Translation in Sepsis-Induced Acute Respiratory Distress Syndrome. JCI Insight (2019) 4(2):e124061. 10.1172/jci.insight.124061 PMC6413834 30674720

[5] Huppert L Matthay M Ware L. Pathogenesis of Acute Respiratory Distress Syndrome. Semin Respir Crit Care Med (2019) 40(1):031–9. 10.1055/s-0039-1683996 PMC7060969 31060086

[6] Eworuke E Major JM Gilbert McClain LI. National Incidence Rates for Acute Respiratory Distress Syndrome (ARDS) and ARDS Cause-specific Factors in the United States (2006-2014). J Crit Care (2018) 47:192–7. 10.1016/j.jcrc.2018.07.002 30015289

[7] Bellani G Laffey JG Pham T Fan E Brochard L Esteban A Epidemiology, Patterns of Care, and Mortality for Patients with Acute Respiratory Distress Syndrome in Intensive Care Units in 50 Countries. JAMA (2016) 315(8):788–800. 10.1001/jama.2016.0291 26903337

[8] Force ADT Rubenfeld GD Thompson BT Ferguson ND Caldwell E Fan E Acute Respiratory Distress Syndrome: the Berlin Definition. JAMA (2012) 307(23):2526–33. 10.1001/jama.2012.5669 22797452

[9] Stanski NL Wong HR. Prognostic and Predictive Enrichment in Sepsis. Nat Rev Nephrol (2020) 16(1):20–31. 10.1038/s41581-019-0199-3 31511662 PMC7097452

[10] Kangelaris KN Prakash A Liu KD Aouizerat B Woodruff PG Erle DJ Increased Expression of Neutrophil-Related Genes in Patients with Early Sepsis-Induced ARDS. Am J Physiol Lung Cell Mol Physiol (2015) 308(11):L1102–L1113. 10.1152/ajplung.00380.2014 25795726 PMC4451399

[11] Zhang J Luo Y Wang X Zhu J Li Q Feng J Global Transcriptional Regulation of STAT3- and MYC-Mediated Sepsis-Induced ARDS. Ther Adv Respir Dis (2019) 13:1753466619879840. 10.1177/1753466619879840 31566109 PMC6769203

[12] Zhang Z Han N Shen Y. S100A12 Promotes Inflammation and Cell Apoptosis in Sepsis-Induced ARDS *via* Activation of NLRP3 Inflammasome Signaling. Mol Immunol (2020) 122:38–48. 10.1016/j.molimm.2020.03.022 32298873

[13] Xue M Sun Z Shao M Yin J Deng Z Zhang J Diagnostic and Prognostic Utility of Tissue Factor for Severe Sepsis and Sepsis-Induced Acute Lung Injury. J Transl Med (2015) 13:172. 10.1186/s12967-015-0518-9 26025445 PMC4459056

[14] Dolinay T Kim YS Howrylak J Hunninghake GM An CH Fredenburgh L Inflammasome-regulated Cytokines Are Critical Mediators of Acute Lung Injury. Am J Respir Crit Care Med (2012) 185(11):1225–34. 10.1164/rccm.201201-0003oc 22461369 PMC3373064

[15] Ritchie ME Phipson B Wu D Hu Y Law CW Shi W Limma powers Differential Expression Analyses for RNA-Sequencing and Microarray Studies. Nucleic Acids Res (2015) 43(7):e47. 10.1093/nar/gkv007 25605792 PMC4402510

[16] Yu G Wang L-G Han Y He Q-Y. clusterProfiler: an R Package for Comparing Biological Themes Among Gene Clusters. OMICS: A J Integr Biol (2012) 16(5):284–7. 10.1089/omi.2011.0118 PMC3339379 22455463

[17] Friedman J Hastie T Tibshirani R. Regularization Paths for Generalized Linear Models via Coordinate Descent. J Stat Softw (2010) 33(1):1–22. 10.18637/jss.v033.i01 20808728 PMC2929880

[18] Sagy M Al-Qaqaa Y Kim P. Definitions and Pathophysiology of Sepsis. Curr Probl Pediatr Adolesc Health Care (2013) 43(10):260–3. 10.1016/j.cppeds.2013.10.001 24295606

[19] Stevens JP Law A Giannakoulis J. Acute Respiratory Distress Syndrome. JAMA (2018) 319(7):732. 10.1001/jama.2018.0483 29466593

[20] Ferrè S Deng Y Huen SC Lu CY Scherer PE Igarashi P Renal Tubular Cell Spliced X-Box Binding Protein 1 (Xbp1s) Has a Unique Role in Sepsis-Induced Acute Kidney Injury and Inflammation. Kidney Int (2019) 96(6):1359–73. 10.1016/j.kint.2019.06.023 31601454 PMC7286357

[21] Li L Dong L Zhao D Gao F Yan J. Classical Dendritic Cells Regulate Acute Lung Inflammation and Injury in Mice with Lipopolysaccharide-Induced Acute Respiratory Distress Syndrome. Int J Mol Med (2019) 44(2):617–29. 10.3892/ijmm.2019.4208 31173158 PMC6605708

[22] Meng L Cao H Wan C Jiang L. MiR-539-5p Alleviates Sepsis-Induced Acute Lung Injury by Targeting ROCK1. Folia Histochem Cytobiol (2019) 57(4):168–78. 10.5603/FHC.a2019.0019 31825519

[23] Sweeney TE Azad TD Donato M Haynes WA Perumal TM Henao R Unsupervised Analysis of Transcriptomics in Bacterial Sepsis Across Multiple Datasets Reveals Three Robust Clusters. Crit Care Med (2018) 46(6):915–25. 10.1097/ccm.0000000000003084 29537985 PMC5953807

[24] Mori S Jewett A Cavalcanti M Murakami-Mori K Nakamura S Bonavida B. Differential Regulation of Human NK Cell-Associated Gene Expression Following Activation by IL-2, IFN-Alpha and PMA/ionomycin. Int J Oncol (1998) 12(5):1165–70. 10.3892/ijo.12.5.1165 9538144

[25] Gao Q Cui Y Shen Y Li Y Gao X Xi Y Identifying Mutually Exclusive Gene Sets with Prognostic Value and Novel Potential Driver Genes in Patients with Glioblastoma. Biomed Res Int (2019) 2019:4860367. 10.1155/2019/4860367 31815141 PMC6878817

[26] Fan C Wang J Mao C Li W Liu K Wang Z. The FGL2 Prothrombinase Contributes to the Pathological Process of Experimental Pulmonary Hypertension. J Appl Physiol (1985) (2019) 127(6):1677–87. 10.1152/japplphysiol.00396.2019 31580221

[27] Lee H-K Park D-W Bae JH Kim HJ Shin D-G Park J-S RGS2 Is a Negative Regulator of STAT3-Mediated Nox1 Expression. Cell Signal (2012) 24(3):803–9. 10.1016/j.cellsig.2011.11.015 22120521

[28] Chiang K-C Huang S-T Wu R-C Huang S-C Yeh T-S Chen M-H Interferon α-inducible Protein 27 Is an Oncogene and Highly Expressed in Cholangiocarcinoma Patients with Poor Survival. Cancer Manag Res (2019) 11:1893–905. 10.2147/cmar.s196485 30881116 PMC6400119

