# Supplementary material for: A Five-Genes Based Diagnostic Signature for Sepsis-Induced ARDS
Source: Pathol Oncol Res. 2021 Jul 29;27:580801. doi: 10.3389/pore.2021.580801 (PMC8357742; doi:10.3389/pore.2021.580801)
Supplement: Supplementary file 1 [file Table1.docx]

Table S1. Clinical characteristics in sepsis and ARDS vs. sepsis alone of GSE66890

| **Characteristics** | **Sepsis Only (*N=*28)** | **Sepsis ARDS (*N=*29)** | ***P* Value** |
| --- | --- | --- | --- |
| Age (mean ± SD) | 67 ± 20 | 59 ± 19 | 0.11 |
| Gender (Male(%)) | 16 (57%) | 16 (55%) | 0.61 |
| PAXgene batch 2 | 17 (61%) | 19 (66%) | 0.71 |
| Time to PAXgene draw after ICU admission, mean hours SD | 9.2 ± 8.1 | 10.9 ± 9.8 | 0.46 |
| WBC, median (IQR), cells/ul | 14.2 (11.2, 18.6) | 11.1 (6.6, 12.6) | 0.007 |
| ANC, median (IQR), cells/ul | 12.1 (8.5, 16.8) | 9.4 (5.0, 11.8) | 0.04 |
| APACHE III, mean SD | 84 ± 31 | 116 ± 39 | 0.002 |
| Shock | 12 (43%) | 21 (72%) | 0.02 |
| 60-day mortality | 5 (18%) | 9 (31%) | 0.25 |

WBC, white blood cell count; ANC, absolute neutrophil count; APACHE, Acute Physiology and Chronic Health Evaluation; ARDS, acute respiratory distress syndrome; ESRD, end-stage renal disease; ICU, intensive careunit; IQR, interquartile range; SD, standard deviation
