# Supplementary figures and images for: A Five-Genes Based Diagnostic Signature for Sepsis-Induced ARDS
Source: Pathol Oncol Res. 2021 Jul 29;27:580801. doi: 10.3389/pore.2021.580801 (PMC8357742; doi:10.3389/pore.2021.580801)

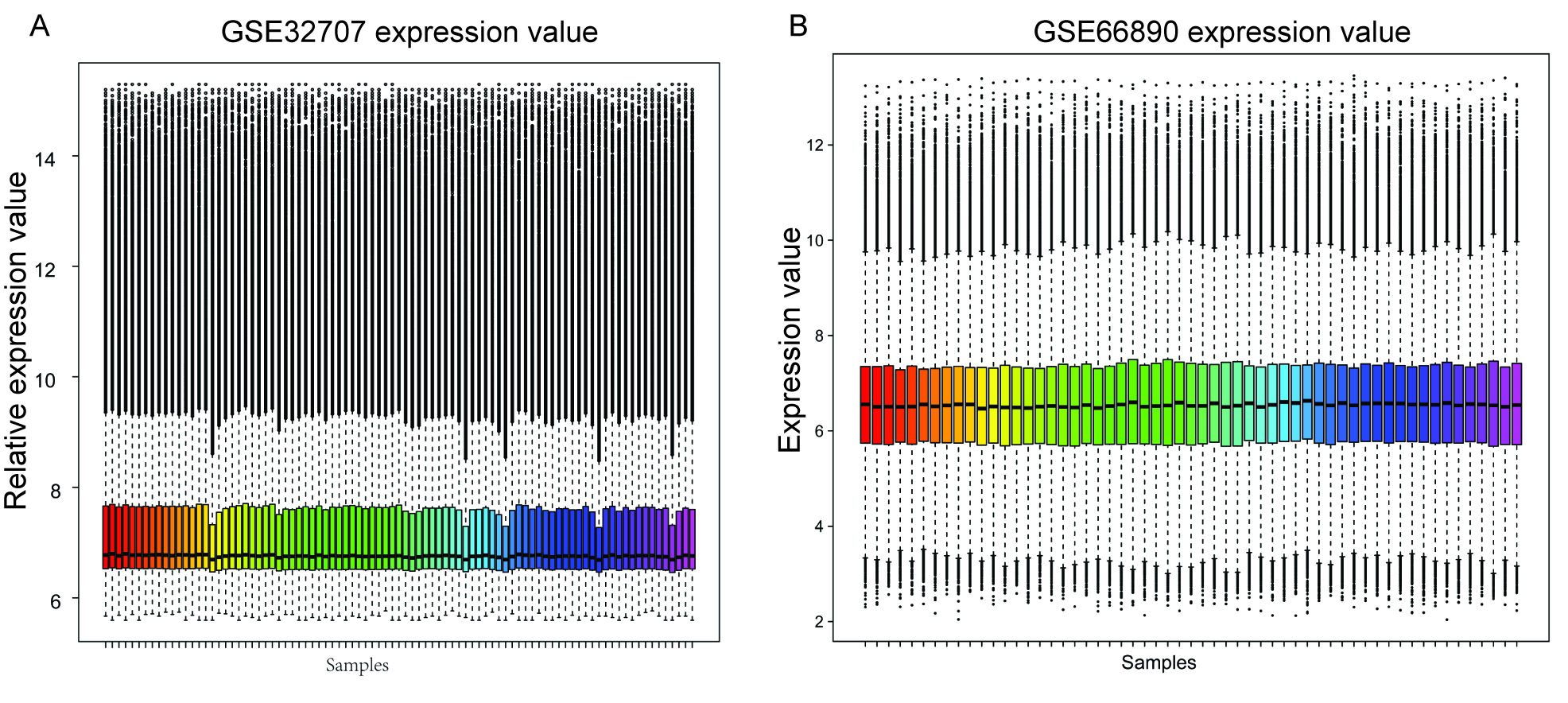

Supplement: Supplementary file 5 [file Image1.TIF]
